# S‐PLM: Structure‐Aware Protein Language Model via Contrastive Learning Between Sequence and Structure

**DOI:** 10.1002/advs.202404212

**Published:** 2024-12-12

**Authors:** Duolin Wang, Mahdi Pourmirzaei, Usman L. Abbas, Shuai Zeng, Negin Manshour, Farzaneh Esmaili, Biplab Poudel, Yuexu Jiang, Qing Shao, Jin Chen, Dong Xu

**Affiliations:** ^1^ Department of Electrical Engineering and Computer Science and Christopher S. Bond Life Sciences Center University of Missouri Columbia MO 65211 USA; ^2^ Chemical & Materials Engineering University of Kentucky Lexington KY 40506 USA; ^3^ Department of Medicine and Department of Biomedical Informatics and Data Science University of Alabama at Birmingham Birmingham AL 35294 USA

**Keywords:** contrastive learning, deep learning, protein function prediction, protein language model, protein structure

## Abstract

Proteins play an essential role in various biological and engineering processes. Large protein language models (PLMs) present excellent potential to reshape protein research by accelerating the determination of protein functions and the design of proteins with the desired functions. The prediction and design capacity of PLMs relies on the representation gained from the protein sequences. However, the lack of crucial 3D structure information in most PLMs restricts the prediction capacity of PLMs in various applications, especially those heavily dependent on 3D structures. To address this issue, S‐PLM is introduced as a 3D structure‐aware PLM that utilizes multi‐view contrastive learning to align the sequence and 3D structure of a protein in a coordinated latent space. S‐PLM applies Swin‐Transformer on AlphaFold‐predicted protein structures to embed the structural information and fuses it into sequence‐based embedding from ESM2. Additionally, a library of lightweight tuning tools is provided to adapt S‐PLM for diverse downstream protein prediction tasks. The results demonstrate S‐PLM's superior performance over sequence‐only PLMs on all protein clustering and classification tasks, achieving competitiveness comparable to state‐of‐the‐art methods requiring both sequence and structure inputs. S‐PLM and its lightweight tuning tools are available at https://github.com/duolinwang/S-PLM/.

## Graphical abstract

S‐PLM is a structure‐aware protein language model (PLM) that integrates protein 3D structure information into sequence‐based embeddings through multi‐view contrastive learning. S‐PLM requires only sequence input during inference, avoiding the need for protein structures. The model outperforms sequence‐only PLMs on various protein prediction tasks and provides a library of lightweight tuning tools for downstream applications.

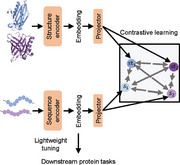

## 1 Introduction

Proteins play a crucial role in resolving many of the health, energy, and environmental challenges facing society today. An essential task is to gain information about proteins of interest quickly and accurately. Along this line, computational predictions of protein properties from protein sequences play important roles. Protein language models (PLMs) can reveal the underlying patterns within protein sequences and predict properties based on the underlying sequence patterns.^[^ ^1^ ^]^ The current paradigm for PLM development and deployment includes two stages: 1) train a PLM to convert the amino acid sequence into a latent representation (embedding) by means of the masked language modeling (MLM) or autoregressive strategy, where the model predicts masked or next amino acids in the sequence based on the surrounding or previous context^[^ ^2^, ^3^, ^4^ ^]^; and 2) adapt the pretrained PLM with the protein property^[^ ^2^ ^]^ data to perform specific protein tasks. Several PLMs have been developed following this paradigm, such as the ProtBert,^[^ ^3^ ^]^ ESM2^[^ ^5^ ^]^ models and ProtGPT2.^[^ ^6^ ^]^ These PLMs have shown encouraging results for protein property predictions and de novo protein designs and demonstrated their potential for new knowledge discovery and analyses.^[^ ^7^, ^8^, ^9^ ^]^

One challenge in developing PLMs is enriching critical biophysical information into the embeddings. It is well known that a protein's function relies on its 3D structure. However, most PLMs are trained solely on amino acid sequences, thereby constraining their predictive capabilities, especially those heavily depending on 3D protein structures. Some methods have been developed to enrich the evolutionary or functional information in the sequence‐based embedding. One method integrates multiple sequence alignments (MSAs) into PLMs, such as AlphaFold's Evoformer^[^ ^10^ ^]^ and MSA Transformer.^[^ ^11^ ^]^ ProteinBERT incorporated gene ontology (GO) annotations into the MLM pretraining scheme.^[^ ^12^ ^]^ It utilized a denoising autoencoder to pretrain the model with corrupted protein sequences and GO annotations and performed comparatively well in many protein tasks despite its relatively smaller size. These methods enriched the information in the sequence‐based embedding and boosted the performance of the PLMs. However, none of these methods incorporated the critical structural information in an indirect manner.

Another emerging method is to develop a joint embedding with both sequence and structural inputs. Chen et al. proposed a self‐supervised learning‐based method for protein structure representation to leverage the available pretrained PLMs.^[^ ^13^ ^]^ Zhang et al. explored the joint embedding for proteins based on ESM2 and three distinct structure encoders.^[^ ^14^ ^]^ Hu et al. also developed a joint embedding for proteins by coupling protein sequence and structure.^[^ ^15^ ^]^ These joint embeddings performed better in numerous protein property prediction tasks, highlighting the importance of including structural information in the protein representation. However, the utilization of these joint‐embedding models requires both sequence and structure as input. Even with prediction tools such as AlphaFold, gaining reliable structures for some specific proteins remains a challenge. Furthermore, computationally, these methods take an extra step to obtain predicted structures, which requires extra time and effort.

We propose an alternative approach to protein representation learning. Instead of creating a joint embedding that needs both sequence and structural inputs, we have developed a sequence‐based embedding that incorporates structural information. One feasible strategy for incorporating structural information into sequence‐based embeddings is through cross‐view representation learning. PromptProtein^[^ ^16^ ^]^ exemplifies this approach by pretraining on a sequence‐to‐structure prediction task to generate structure‐aware representations. In contrast, our approach is based on another technique known as multi‐view contrastive learning. Unlike cross‐view representation learning, which translates embeddings from one view to another, multi‐view contrastive learning aligns embeddings into a coordinated latent space across multiple views. This technique has garnered significant attention for its ability to capture rich and complementary features from diverse perspectives.^[^ ^17^, ^18^ ^]^ During training, the contrastive loss function pushes the representations of similar views (sequence and structure of the same protein) to be close to each other in the embedding space while simultaneously separating representations of dissimilar views (between different proteins) further apart. As a result, multi‐view contrastive learning effectively captures underlying semantic patterns in the data. A head‐to‐head comparison has demonstrated that multi‐view contrastive learning can be more effective than cross‐view representation learning.^[^ ^18^ ^]^

To this end, we propose S‐PLM, a 3D structure‐aware PLM that enables the sequence‐based embedding to carry the structural information through multi‐view contrastive learning. One advantage of S‐PLM compared to those joint‐embedding models is that after training, S‐PLM only needs amino acid sequences as the input during inference. This sequence‐only input avoids the overhead of using predicted protein structures. It is worth mentioning that two other recent successful cases have illustrated the potential of contrastive learning in enriching information in sequence‐based embedding. One is the ProtST, which injected biomedical text into a PLM by aligning these two modalities through a contrastive loss.^[^ ^19^ ^]^ After training, their PLM contained enhanced protein property information and demonstrated superiority over previous models on diverse protein representations and classification benchmarks. Another one is the CLEAN^[^ ^20^ ^]^ model, which utilized information from the EC number to enhance the ability of PLM to predict enzymatic functions based on protein sequence. Although S‐PLM can accommodate either structure or sequence as the input, this paper will only demonstrate the advantages of S‐PLM in encoding protein sequences and leveraging structural information to improve sequence representations for various protein prediction tasks. The sequence encoder of S‐PLM was implemented based on a pretrained ESM2 model, offering extensibility to incorporate new protein properties incrementally without forgetting previous knowledge in the model.^[^ ^21^ ^]^

To apply pretrained PLMs to specific protein prediction tasks, a typical domain adaptation approach involves fine‐tuning the PLMs on domain‐specific data to update all model parameters. However, full fine‐tuning may not work well for large PLMs due to limited domain‐specific training data that may lead to catastrophic forgetting^[^ ^22^ ^]^ or severe computational and memory costs. To address this challenge, researchers have developed a set of lightweight tuning strategies, making specific and parameter‐efficient modifications to large preexisting models. These strategies, such as fine‐tuning top layers, adapter tuning,^[^ ^21^ ^]^ and low‐rank adaptation (LoRA),^[^ ^23^ ^]^ selectively update targeted parameters while keeping others frozen, significantly reducing computational and memory requirements and mitigating data scarcity while achieving comparable or superior performance. However, there was limited research on applying these methods to PLMs.^[^ ^24^, ^25^ ^]^ This work explored several lightweight tuning strategies for diverse protein prediction tasks utilizing S‐PLM. Through further lightweight tuning, S‐PLM achieved competitive performance in gene ontology,^[^ ^26^ ^]^ enzyme commission number,^[^ ^27^ ^]^ and protein secondary structure prediction tasks. A library of lightweight tuning methods has been made available at https://github.com/duolinwang/S-PLM/.

## 2 Results

### 2.1 Structure‐Aware Protein Language Model (S‐PLM)

**Figure 1:**
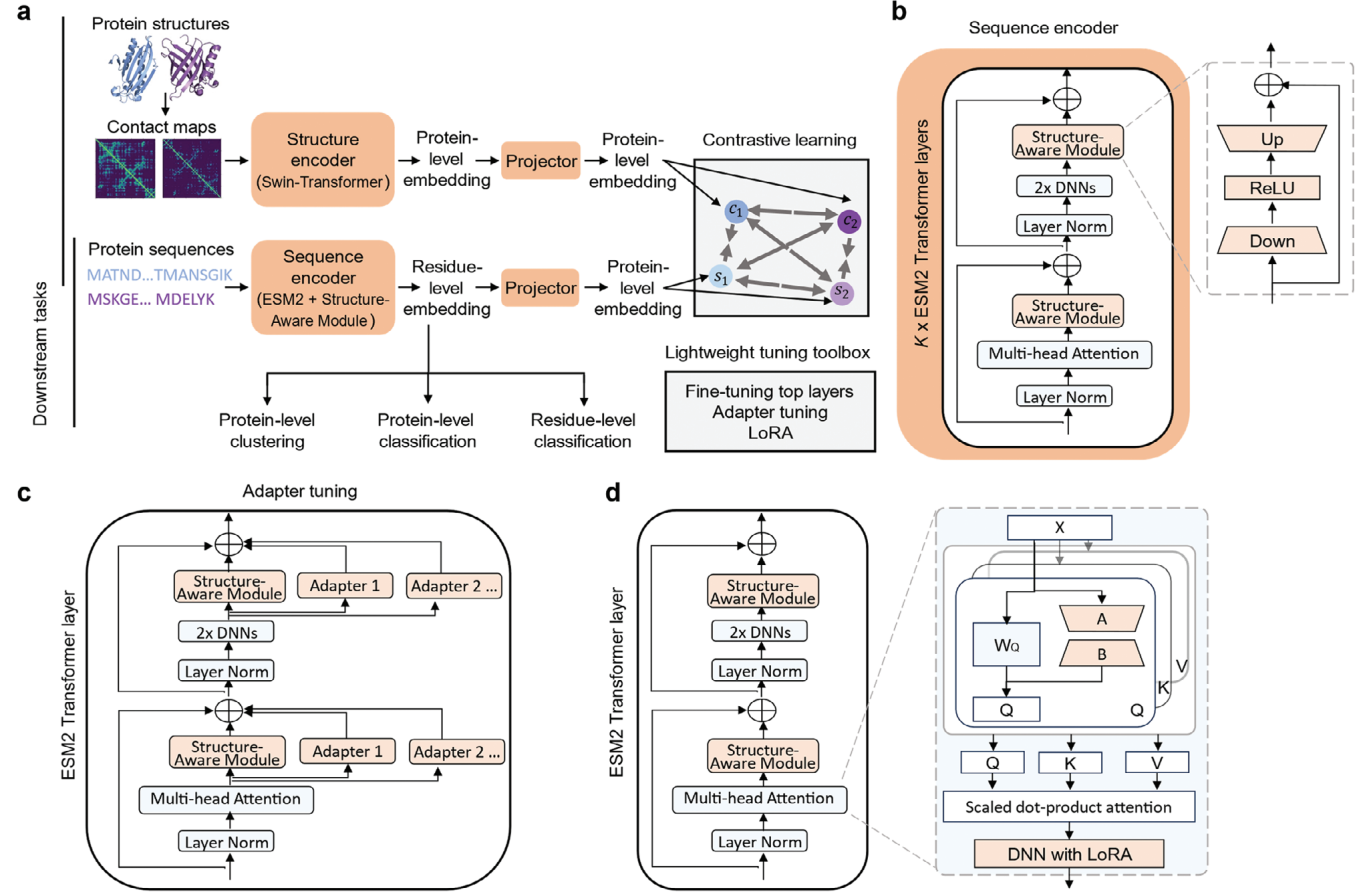
The framework of S‐PLM and lightweight tuning strategies for downstream supervised learning. a) The framework of S‐PLM. During pretraining, the model inputs the amino acid sequences and contact maps simultaneously. The amino acid sequences of proteins are converted into residue‐level embeddings through a sequence encoder (ESM2+Structure‐Aware Module), while the backbone C_α_ contact maps of proteins are converted into protein‐level embeddings through a structure encoder (Swin‐Transformer). With respective projectors, the S‐PLM model is trained through contrastive learning on the protein‐level embeddings from each modality. After pretraining, the sequence encoder that generates the residue‐level embeddings before the projector layer is used for downstream tasks, such as protein‐level clustering, classification, and residue‐level classification tasks. b) Architecture of the sequence encoder. The sequence encoder of S‐PLM is tuned on the top‐*K* Transformer layers of ESM2, each adding a compact Structure‐Aware Module. During the pretraining, the ESM2 backbone model is frozen, and only the Structure‐Aware Modules are trainable. c) Adapter tunning for downstream supervised tasks is implemented by integrating additional paralleled adapters on the ESM2 transformer layers. d) LoRA tuning for downstream supervised tasks is implemented by adding trainable rank decomposition matrices into the multi‐head attention layer of top‐*K* Transformer layers in ESM2.

**Figure** **1**a depicts the framework of S‐PLM. The pretraining architecture of S‐PLM consists of two encoders, one to encode protein sequences and the other to encode 3D protein structures. In this study, the one‐letter amino acid sequences are utilized as the input of protein sequences. The backbone C_α_ contact maps are used to represent the protein 3D structures because inter‐residue distances contain comprehensive and essential information about protein structure. During pretraining, S‐PLM inputs both amino acid sequences and backbone C_α_ contact maps. The protein sequence information is converted into the residue‐level embedding through a sequence encoder, while the contact map information is transformed into a protein‐level embedding through a structure encoder (Swin‐Transformer^[^ ^28^ ^]^). Then through each corresponding projector, sequences and contact maps are converted into separate protein‐level embeddings (Methods 1 and 2). Finally, the S‐PLM model is trained using contrastive learning to minimize the contrastive loss for a batch of sequences and contact maps. The objective of the S‐PLM model is to maximize the alignment of the embeddings for the sequence and structure from the same protein and clearly separate the embeddings for the sequences and structures between different proteins (de‐alignment). Inspired by the SimCLR method,^[^ ^29^ ^]^ our work adapts the CLIP^[^ ^30^ ^]^ approach for contrastive language‐image pretraining. Besides the CLIP's alignment and de‐alignment across different modalities, our model also accounts for de‐alignment within the same modality. For instance, as shown in Figure 1a, our model also emphasizes dissimilarity between embeddings of sequences (*s*_1_↔*s*_2_) and embeddings of contact maps (*c*_1_↔*c*_2_) from different proteins (Methods 3).

The sequence encoder of S‐PLM was implemented by incorporating a Structure‐Aware Module into the ESM2 model (Figure 1b). There are several approaches to implementing this Structure‐Aware Module. One option is to fine‐tune the ESM2 model by adjusting its pretrained weights, which retains the original architecture of the ESM2 encoder while integrating structural information. Alternatively, adapter tuning^[^ ^21^ ^]^ can be employed, where adapter modules are integrated into the top‐*K* Transformer layers of the ESM2 model. These adapter modules serve as the structure‐aware component and are only trained during the pretraining process. As shown in Figure 1b, the Structure‐Aware Modules consist of a bottleneck structure and a skip‐connection, positioned twice in one Transformer layer: after the multi‐head attention projection and after the two feed‐forward layers (Methods 5). Using adapter tuning to implement the sequence encoder of S‐PLM offers several advantages. First, the integrated adapter module is compact. It contains much fewer parameters than the original Transformer modules of ESM2, which alleviates the training burden. Second, it allows for continuous training to add new protein features (e.g., protein function) for the future extension of our model, without catastrophic forgetting of previously learned features, because the S‐PLM pretraining retains the sequence representation capabilities of the ESM2 model with the ESM2 backbone model (the architecture and its weights) intact.

During the inference stage, S‐PLM has the flexibility to accept either sequence or contact map as the input and produces corresponding embeddings at various levels tailored to specific downstream tasks (e.g., protein‐level clustering, protein‐level classification, and residue‐level classification). This versatility allows S‐PLM to adapt and provide suitable representations based on the specific input data and requirements of the given task. In this paper and the subsequent results, the S‐PLM model primarily generates sequence embeddings from protein sequences. Therefore, the pre‐trained sequence encoder of S‐PLM that generates the residue‐level embeddings before deploying projectors is used for downstream tasks. The entire sequence encoder can be fully frozen or learnable. To fully exploit the potential of S‐PLM in supervised protein prediction tasks, we have developed several lightweight tuning strategies based on the sequence encoder of S‐PLM, all of which are incorporated into the lightweight tuning toolbox, including the fine‐tuning top layers, adapter tuning (Figure 1c), and LoRA tuning (Figure 1d) (Methods 5).

### 2.2 Contrastive Learning Rearranges the Alignment Between the Sequence and Structure Embeddings

**Figure 2:**
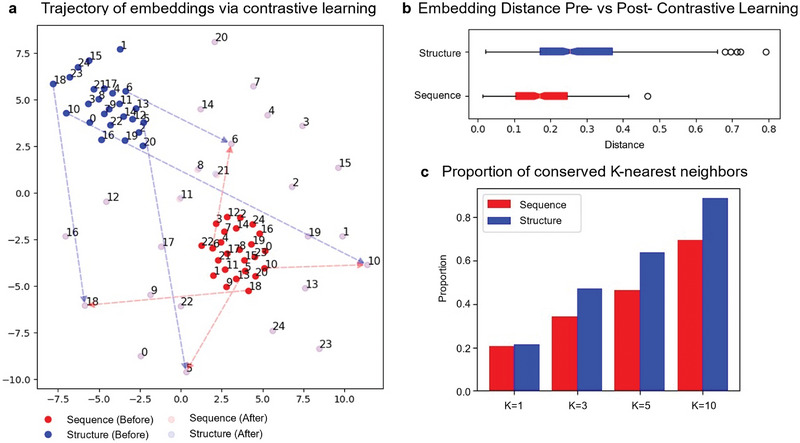
Structure and sequence embedding rearrangement through contrastive learning. a) Trajectories illustrating the alignment of sequence and structure embeddings via contrastive learning. Blue nodes indicate structure‐based 2D‐t‐SNE embeddings for proteins, whereas red nodes indicate sequence‐based 2D‐t‐SNE embeddings for proteins. The number beside each node indicates the protein index (from 0 to 24). The trajectories of four randomly selected nodes (5, 6, 10, 18) before and after the contrastive learning are displayed. The trajectories for other nodes have the same pattern (omitted for clear illustration). After contrastive learning, the sequence and structure embeddings of the same protein are almost identical, resulting in overlapping solid circles. b) Distance distribution between sequence or structure embeddings before and after contrastive learning. Each distance is calculated using the Euclidean distance between a sequence or structure embedding before contrastive learning and the corresponding embedding of the same protein after contrastive learning in the original embedding space. c) Proportion of conserved *K*‐nearest neighbors. Each bar represents the proportion of conserved *K*‐nearest neighbors for a specific value of *K*. Five hundred proteins were used for the analyses in panels (b,c).

We investigated the impact of contrastive learning on the alignment between sequence and structure embeddings. Initially, we randomly selected 25 proteins from an independent test set (500 proteins in total) and projected their corresponding sequence and structure embeddings onto a 2D t‐SNE space. We employed trajectory plots of sequence and structure embeddings to illustrate the movement of embeddings during contrastive learning. As depicted in **Figure** **2**a, before contrastive learning, the sequence‐based embeddings (obtained from pretrained ESM2‐t33_650M_UR50D) and the structure‐based embeddings (obtained from pretrained Swin‐Transformer) were distinctly distributed in the 2D t‐SNE space. Embeddings from the same modality were closer than those from different modalities, indicating no information exchange between the two modalities. Conversely, after contrastive learning, sequence and structure embeddings of the same protein moved closer, whereas those of different proteins became more separated, suggesting successful integration of protein structure information into sequence‐based embeddings (and vice versa). To show the effect of protein size in our training strategy, we grouped the proteins in the test dataset into different bins based on their sequence length. Figure S1 (Supporting Information) shows the embedding distributions after contrastive learning within specific sequence length ranges. This experiment shows that the observed alignment is not influenced by sequence length.

Notably in Figure 2a, the changes in structure embeddings were more pronounced than those in sequence embeddings, causing them to spread around the original embeddings. This observation is further supported by Figure 2b, which shows the distribution of embedding distances for each modality across the entire 500 testing proteins before and after contrastive learning, revealing more significant embedding changes in structure than in sequence. This phenomenon also underscores that our sequence encoder of S‐PLM effectively retained the sequence representation capabilities of the base ESM2 model, minimizing catastrophic forgetting. Additionally, we quantitatively assessed the effectiveness of contrastive learning in preserving the neighborhood topology and relationships within sequence and structure embeddings by calculating the proportion of conserved K‐nearest neighbors for each modality after contrastive learning across different values of *K* (Figure 2c). For the entire set of 500 testing proteins, over 20% of the first‐nearest neighbors of both the sequence and structure embeddings were preserved after contrastive learning. Increasing *K* to 10 preserved 69% of the sequence embedding neighbors and 89% of the structure embedding neighbors. Across all *K* values, the proportions of preserved structure embedding neighbors were higher than those of sequence, indicating that our training process effectively preserved more topological and geometrical information within the structure embeddings.

### 2.3 S‐PLM Injects the Structural Information into the Sequence of Latent Space

**Figure 3:**
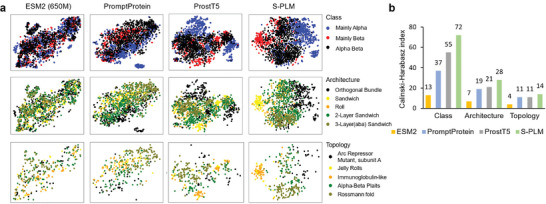
Visualization and benchmark of protein embeddings for three CATH structural hierarchies (class, architecture, and topology). a) t‐SNE visualization of protein embeddings from the five most represented categories from one hierarchy. Embeddings produced by ESM2, PromptProtein, and ProstT5 are shown side by side with S‐PLM. b) Utilizing the CHI to quantitatively assess the capability of embeddings derived from different methods in clustering CATH structural categories.

To investigate whether S‐PLM can inject the structural information into the sequence latent space, we evaluated the sequence representations for the CATH protein domains.^[^ ^31^ ^]^ Because this experiment prioritizes structural information, we deliberately chose a single representative sequence from each CATH superfamily to provide clear visualization. We utilized the CATHS40 dataset, whose proteins have a maximal 40% sequence identity. Our analysis focused on the class, architecture, and topology levels of the CATH hierarchy, excluding the homologous superfamily at the last level, which comprises clusters primarily driven by sequence similarity. We visualized and benchmarked the sequence representations produced by S‐PLM against models that rely solely on sequence information, including ESM2 (ESM2_t33_650M_UR50D); the pretrained PLM on which S‐PLM is based; PromptProtein^[^ ^16^ ^]^ and ProstT5,^[^ ^32^ ^]^ other two structure‐aware models pretrained by predicting 3D structures or 3D structure tokens from sequences (**Figure** **3**a). Each row of Figure 3a shows the t‐SNE visualization of protein embeddings from the five most represented categories of one hierarchy. It shows that sequence representation from S‐PLM separates CATH structural classes more clearly than the embeddings from other models.

We further utilized the Calinski–Harabasz index (CHI)^[^ ^32^ ^]^ to quantitatively assess the capability of embeddings derived from different methods in distinguishing CATH structural categories. The CHI score quantifies the ratio between the sum of between‐cluster dispersion and the sum of within‐cluster dispersion. We applied the CATH categories to define ground‐truth clusters, using sequence embeddings to calculate both between‐ and within‐cluster dispersion. As shown in Figure 3b, the CHI of S‐PLM is around 30% higher than the second (ProsT5) for Class, Architecture, and Topology, and around 300% higher than ESM2. Given that these CATH categories were established using protein structures, this analysis strongly suggests that the sequence embedding produced by the developed S‐PLM exhibits an inherent awareness of protein structures, surpassing the other two PLMs in effectively distinguishing proteins with diverse structural characteristics. The poor performance of the sequence‐only ESM2 also indicated its limitations in explicitly acquiring protein structure knowledge.

### 2.4 Clustering Enzymes via S‐PLM

**Figure 4:**
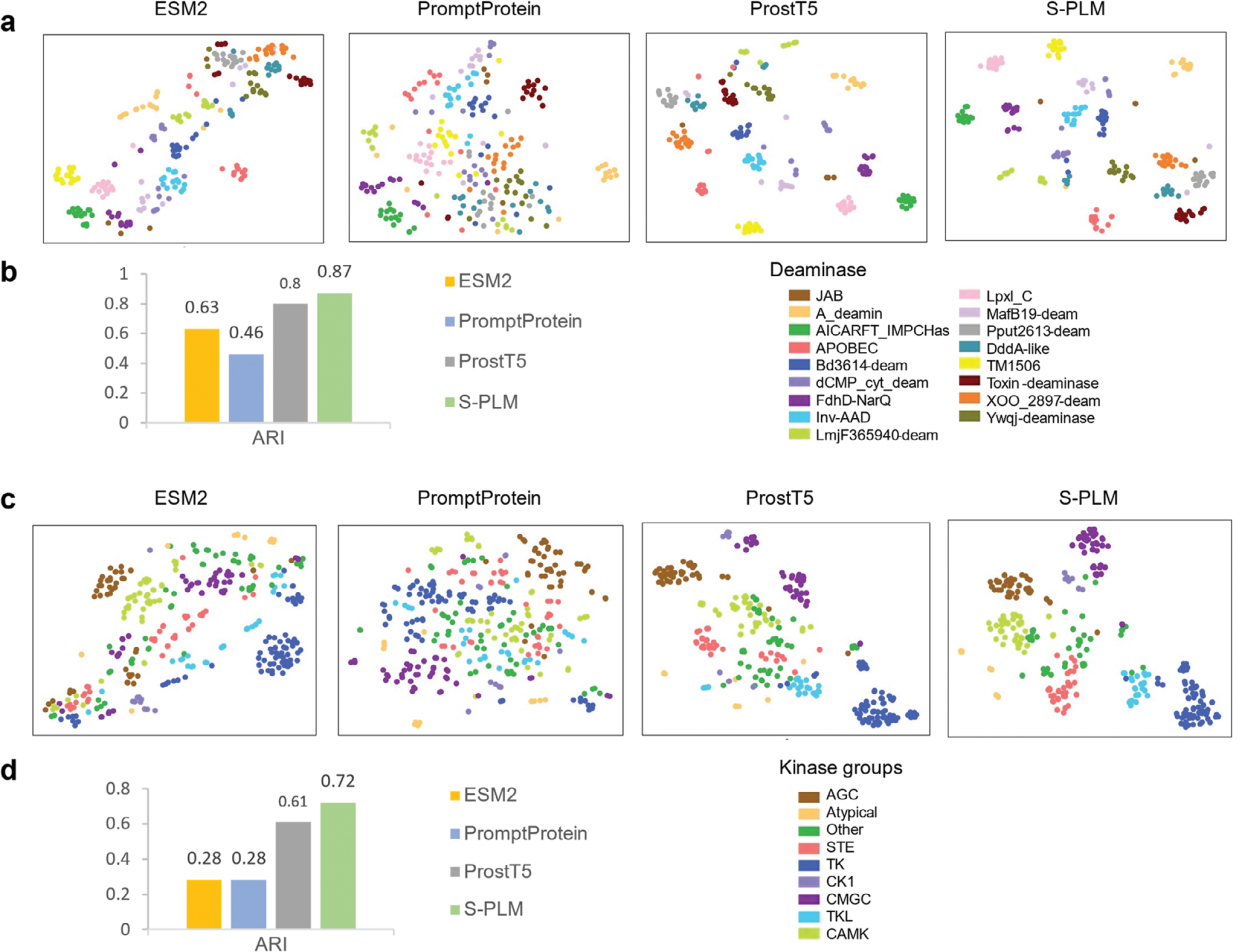
Clustering enzymes. a) The t‐SNE visualization of protein embeddings from 242 deaminase proteins using sequence representation methods of ESM2, PromptProtein, ProstT5, and S‐PLM. b) Quantitative benchmarks of PLM's ability to cluster the deaminase proteins for sequence representation methods. c) The t‐SNE visualization of protein embeddings from human kinase groups using pretrained PLM models of ESM2, PromptProtein, ProstT5, and S‐PLM. Different kinase groups are distinguished by different colors. d) Quantitative benchmarks of PLM's ability to cluster the human kinases compared with other methods mentioned in (c). For both types of enzymes, ARI was computed by comparing K‐Means clustering assignments with enzyme labels.

Recently, Huang et al. applied a structure‐based protein clustering approach for discovering deaminase functions and identifying novel deaminase families.^[^ ^33^ ^]^ They applied AlphaFold2 to predict protein structures and subsequently clustered the entire deaminase protein family based on the predicted structure similarities through structure alignment. They discovered new functions of the deaminase proteins and new deaminases; such findings cannot be obtained by mining amino acid sequences. In this study, we investigated the effectiveness of our S‐PLM model in clustering the deaminase family by comparing and benchmarking the sequence embeddings from S‐PLM against the other three PLMs, i.e., the ESM2, PromptProtein, and ProstT5. We utilized the same sequence data as Huang et al. to generate representations for each query protein sequence. For this task, we utilized the S‐PLM and obtained a 1280‐dimensional embedding for each protein. Subsequently, we employed t‐SNE to reduce the vector to a 2D representation and applied the K‐Means clustering method to the reduced dimension. The Adjusted Rand Index (ARI) was computed by comparing K‐Means clustering assignments with known deaminase family annotations. We conducted the t‐SNE visualization and calculated the ARI in the same way for all the benchmarked methods. Based on the comparison (**Figure** **4**a,b), S‐PLM (ARI: 0.87) surpassed the performance of the other three PLMs (ARI: 0.63 for ESM2, 0.46 for PromptProtein, and 0.80 for ProstT5).

We also studied another enzyme group, kinases, which facilitate the transfer of phosphate groups to proteins in a critical process known as phosphorylation.^[^ ^34^ ^]^ We extracted 336 kinases, categorized into nine kinase groups, along with their respective kinase domain sequences from GPS5.0.^[^ ^35^ ^]^ Subsequently, sequence embeddings were generated for each kinase using their corresponding kinase domain sequences. For comparison, we also obtained sequence embeddings from the other three PLMs, ESM2 (ESM2‐t33_650M_UR50D), PromptProtein, and ProstT5. From the t‐SNE plots (Figure 4c) and the ARI comparison (Figure 4d), the sequence embeddings produced by our S‐PLM showed superior clustering of kinase groups, with a significantly higher ARI (0.72) compared to ESM2 (0.28), PromptProtein (0.28), and ProstT5 (0.61). This is likely because the S‐PLM model incorporates protein structure information essential for characterizing kinase groups. Taken together, S‐PLM provides effective sequence representation for enzyme clustering.

### 2.5 S‐PLM Outperforms ESM2 for Protein Fold and Enzyme Reaction Classification

To highlight the performance improvements brought by the structure‐aware module of S‐PLM over its base model ESM2‐650M, we compared S‐PLM with its base model on two structure‐dominated tasks, including the protein fold and enzyme reaction classifications. The same classification layer was applied on top of each encoder, respectively. The comparison comprises two scenarios. In the first scenario, we treated both S‐PLM and ESM2 as protein representation models, freezing all parameters of each encoder and allowing only the classification layers to be trainable. This approach ensures a fair comparison, as the trainable layers in both S‐PLM and ESM2 have the same architecture. In the second scenario, we fine‐tuned the top‐*K* layers of both the S‐PLM and ESM2 models, allowing the encoders to be trainable along with the classification layers. It is important to note that, given the different architectures of S‐PLM and ESM2, achieving absolute fairness in this comparison is impossible, as the optimal configurations for both models may differ. Consequently, to align more closely with ESM2, all configurations were preselected based on ESM2's optimal performance, including the experiments in the first scenario. For scenario two, the key hyperparameter of ESM2 is *K*, (i.e., how many top Transformer layers are trainable). For fold classification, we tried *K* = 5,6,7, and for enzyme reaction, we tried *K* = 1,2. Details regarding the model configurations can be found in Table S1 (Supporting Information).

**Table** **1** shows that the proposed S‐PLM model consistently outperformed the base ESM2 models for all the classification tasks when all encoder layers were frozen (ESM2‐fix vs. S‐PLM‐fix). When an equivalent number of Transformer layers were fine‐tuned (ESM2‐finetune top‐*K* vs. S‐PLM‐finetune top‐*K*), S‐PLM‐finetune outperformed in all tasks except for family classification. Despite a slight 0.2% decrease in family classification, S‐PLM‐finetune demonstrated a notable 2–3% improvement in superfamily and fold predictions. This finding highlights the significance of structural information in fold and superfamily predictions, whereas sequence information prevails in family prediction. We noted that the superior performance of S‐PLM‐finetune does not stem from more trainable parameters compared to ESM2‐finetune. For instance, S‐PLM‐finetune top5 (116M) has fewer parameters than ESM2‐finetune top6 (120M), yet it outperformed in fold (37.74% vs 36.63%) and superfamily (77.59% vs 75.84%) predictions. In enzyme reaction classification, our model achieved 86.71% test accuracy, outperforming ESM2‐finetune top2 (84.50%) with fewer parameters (23M vs 40M). Taken together, these results support the benefits of integrating structural information into our S‐PLM model, particularly for improving predictions in protein fold and enzyme reaction classifications, where structure features are crucial.

**Table 1:** Comparison between S‐PLM and ESM2 for protein fold and enzyme reaction classification.

Method	Protein classification			^a)^Params
	Fold	Superfamily	Family	
ESM2‐fix	27.86	57.26	98.11	1M
S‐PLM‐fix	**34.72**	**62.50**	**98.36**	1M
ESM2‐finetune top5	34.96	76.24	**98.74**	99M
S‐PLM‐finetune top5	**37.74**	**77.59**	98.51	116M
ESM2‐finetune top6	36.63	75.84	**98.82**	120M
S‐PLM‐finetune top6	**37.33**	**77.51**	98.35	139M
ESM2‐finetune top7	36.49	76.56	**98.66**	140M
S‐PLM‐finetune top7	**37.19**	**77.99**	98.43	162M

Method	Enzyme reaction	^a)^Params
ESM2‐fix	78.84	0.5M
S‐PLM‐fix	**82.11**	0.5M
ESM2‐finetune top1	86.53	20M
S‐PLM‐finetune top1	**86.71**	23M
ESM2‐finetune top2	84.50	40M
S‐PLM‐finetune top2	**86.02**	46M

The performance is evaluated by accuracy (%).

^a)^ Params represent the number of trainable parameters. Performance is evaluated by accuracy (%). *K* in “top*K*” indicates how many top Transformer layers are trainable.

### 2.6 Lightweight Tuning Strategies Enhance S‐PLM's Performance in Selected Protein Prediction Tasks

To adapt S‐PLM for various specific supervised protein prediction tasks, we developed lightweight tuning strategies, including fine‐tuning top layers, adapter tuning (Figure 1c), and LoRA (Figure 1d), on the sequence encoder of S‐PLM. We benchmarked S‐PLM with these lightweight tuning strategies on the GO term, EC number, and secondary structure predictions (SS) with state‐of‐the‐art (SOTA) methods. We employed the same training, validation, and test sets for all these tasks, and the only differences are our models and the training strategies. The GO term and EC number predictions served as protein‐level supervised classification tasks, evaluated in terms of the F_max_, while the SS prediction served as the residue‐level supervised classification task evaluated by accuracy (%). Comprehensive results across all three training strategies, along with detailed model descriptions, are provided in **Table** **2**. For GO and EC number predictions, seven methods—GVP,^[^ ^14^ ^]^ GearNet,^[^ ^36^ ^]^ GearNet‐Edge,^[^ ^36^ ^]^ CoupleNet,^[^ ^15^ ^]^ ESM‐GearNet,^[^ ^14^ ^]^ PST (finetuned),^[^ ^37^ ^]^ and SaProt^[^ ^38^ ^]^—that use both sequence and structure inputs, as well as sequence‐only methods ESM2 (650M)^[^ ^14^ ^]^ and ESM‐S,^[^ ^39^ ^]^ were considered for comparison. For the SS task, sequence‐only methods, TAPE (Transformer),^[^ ^40^ ^]^ ProteinBERT,^[^ ^12^ ^]^ ESM‐1b,^[^ ^41^ ^]^ and DML^[^ ^42^ ^]^ were considered for comparison. Their results were taken directly from their respective publications.

**Table 2:** Comparing different lightweight tuning methods for sequence encoders of S‐PLM on GO, EC, and SS prediction with their key model descriptions.

Method	GO (F_max_)			^a)^Params	Sequence encoder layers	Classification layer
	BP	MF	CC			
S‐PLM‐finetune	0.470	0.674	0.460	42M,40M,40M	Finetune top 2 layers, freeze adapter	mean pooling over all residues
ESM2‐finetune	0.473	0.677	0.450			
S‐PLM‐adapter	**0.495**	**0.685**	**0.484**	55M,53M,53M	Adapter tuning top 16 layers	
ESM2‐adapter	0.493	0.683	0.472			
S‐PLM‐LoRA	0.480	0.668	0.472	3M,1M,0.7M	LoRA on top 16 layers, *r* = 2, α = 8
ESM2‐LoRA	0.478	0.663	0.474			

Method	EC (F_max_)	#Params	Sequence encoder layers	Classification layer
S‐PLM‐finetune	0.885	40M	Finetune top 2 layers, freeze adapter	mean pooling over all residues
ESM2‐finetune	0.888			
S‐PLM‐adapter	0.875	53M	Adapter tuning top 16 layers	
ESM2‐adapter	0.866			
S‐PLM‐LoRA	**0.888**	1M	LoRA on top 33 layers, *r* = 2, α = 8	
ESM2‐LoRA	0.864			

Method	SS (Accuracy%)	#Params	Sequence encoder layers	Classification layer
S‐PLM‐finetune	**87.48**	92M	Finetune top 2 layers and all adapter layers	MLP (1280,100,3) on each residue
ESM2‐finetune	87.21			
S‐PLM‐adapter	87.40	52M	Adapter tuning top 16 layers	
ESM2‐adapter	86.73			
S‐PLM‐LoRA	85.72	0.8M	LoRA on top 33 layers, *r* = 2, α = 8	
ESM2‐LoRA	87.14			

^a)^ Params represent the number of trainable parameters.

**Figure 5:**
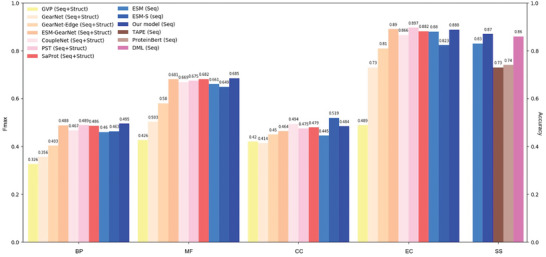
Benchmarking on GO, EC, and SS prediction. Protein level tasks (BP, MF, CC, and EC); residue level task (SS). Seq refers to the use of protein sequences as input, while Struct refers to the use of protein structures as input. Seven methods that use both sequence and structure inputs (GVP,^[^ ^14^ ^]^ GearNet,^[^ ^36^ ^]^ GearNet‐Edge,^[^ ^36^ ^]^ CoupleNet,^[^ ^15^ ^]^ ESM‐GearNet,^[^ ^14^ ^]^ PST finetuned,^[^ ^37^ ^]^ and SaProt^[^ ^38^ ^]^), as well as sequence‐only methods ESM2 (650M)^[^ ^14^ ^]^ and ESM‐S^[^ ^39^ ^]^ were considered for comparison on GO and EC tasks. For the SS task, sequence‐only methods, TAPE (Transformer),^[^ ^40^ ^]^ ProteinBERT,^[^ ^12^ ^]^ ESM‐1b,^[^ ^41^ ^]^ and DML^[^ ^42^ ^]^ were considered for comparison. The best and the second‐best results for each task are shown in bold and underlined symbols, respectively. We used “our model” to represent the performance of S‐PLM with the most effective lightweight tuning strategy tailored to each task.

Upon comparison as shown in **Figure** **5**, our model demonstrated optimum performance in GO‐BP (F_max_: 0.495), GO‐MF (F_max_: 0.686), and SS (F_max_: 87.48) tasks, while exhibiting comparable performance in GO‐CC (F_max_: 0.494 for CoupleNet, 0.519 for ESM‐S, and 0.484 for our model) and in EC number prediction (F_max_: 0.890 for ESM‐GearNet, 0.897 for PST, and 0.888 for our model). Among the methods under comparison, GVP and GearNet are designed to capture the invariant and equivariant features of protein structure, whereas GearNet‐Edge is a variant of GearNet enhanced with an edge message passing layer. CoupleNet requires the integration of sequence and structure information by deeply coupling them, and ESM‐GearNet is a recently proposed variant of GearNet that integrates the sequence representation from PLM with the structure representation through various fusion schemes, where the results with the best fusion scheme were reported in the comparison. SaProt is a newly introduced structure‐aware PLM that explicitly utilizes a structure‐aware vocabulary to integrate residue tokens with structure tokens derived from protein sequence and structure inputs. In contrast to these methods, our model relies solely on protein sequences. Remarkably, it achieves compatible results to CoupleNet, ESM‐GearNet, and PST, or even superior results compared to GVP, GearNet, GearNet‐Edge, and SaProt, showcasing the broader practical utility of our model.

Additionally, by employing the same lightweight tuning strategies on the base ESM2 encoder with an equal number of trainable parameters, we established a fair comparison between our model and its corresponding base model. The results are presented in Table 2. In most (10/15) of the one‐to‐one comparisons, our S‐PLM encoder outperformed its base ESM2 encoder. Although we could not surpass ESM2 across all tasks under all training strategies, our S‐PLM model consistently achieved the best performance overall for each individual task. Overall, our additional Structure‐Aware Module on ESM2, along with the new pretraining process, did not diminish ESM2's original sequence representation capability but rather led to superior performance across all tasks.

### 2.7 Benchmarking Protein Representation Methods on PROBE

The protein representation benchmark (PROBE)^[^ ^43^ ^]^ provides a comprehensive evaluation of protein representation methods by assessing their predictive performance across four independent tasks: semantic similarity inference (SSI), ontology‐based protein function prediction (PFP), drug target protein family classification (PFC), and protein‐protein binding affinity estimation (PPI). The SSI task is unsupervised learning. It measures how well representation models capture biomolecular functional similarity by comparing the calculated similarities between protein vectors using Manhattan distance with the ground truth‐functional similarities derived from GO annotations. Spearman rank‐order correlation values between the vector similarities and GO‐based semantic similarities were used to assess the success of different protein representation methods. The PPI task focuses on the model's ability to predict changes in binding affinities due to residue‐level mutations. The ontology‐based PFP and the drug‐target PFC tasks are addressed using supervised learning, where a linear support vector classifier is trained on labeled data. The PFP task assesses the model's ability to predict protein functions, while the PFC task evaluates the accuracy of classifying drug target proteins into their respective families, which involves proteins with distinct structural features. As mentioned in the PROBE paper,^[^ ^43^ ^]^ the drug target PFC tasks should also show how well the representation can learn structural properties.

In this study, we applied PROBE to evaluate protein representations generated by three sequence‐only, structure‐aware methods: S‐PLM, PromptProtein, and ProstT5, alongside our base model, ESM2‐650M. We also introduced a combined representation, S‐PLM & ESM2‐650M, by averaging the representations from S‐PLM and ESM2‐650M. To streamline the analysis, we only cite the methods from the pre‐generate results that achieved the highest scores in at least one task or subtask, with all the results summarized in **Table** **3**, which aligns with Table 2 in the PROBE paper.

**Table 3:** Benchmark representation methods and their respective predictive performance.

Methods	Semantic similarity inference (based on the Manhattan distance)			Ontology‐based protein function prediction			Drug target protein family classification				PPI binding affinity estimation
	Spearman correlation			Weighted F1‐score			MCC (average)				MSE (average)
	MF	BP	CC	MF	BP	CC	Random	50%	30%	15%	
PFAM^a)^	0.35	0.42	**0.51**	0.86	0.56	0.58	0.90	0.90	0.90	0.81	2.26
ESM‐1b^a)^	0.38	0.42	0.37	0.83	0.53	0.61	0.87	0.84	0.92	0.86	0.48
ProtALBERT^a)^	0.22	0.37	0.32	0.89	0.63	0.64	**0.92**	0.91	0.92	0.88	**0.42**
ProtT5‐XL^a)^	**0.57**	0.21	0.40	**0.90**	**0.66**	**0.68**	**0.92**	**0.92**	0.92	**0.90**	0.6
Mut2Vec^a)^	0.55	**0.58**	0.39	0.57	0.43	0.46	0.44	0.45	0.44	0.46	NA
Prompt Protein	0.31	0.12	0.34	0.77	0.51	0.59	0.82	0.82	0.84	0.79	0.55
ProstT5	0.44	0.09	0.42	0.87	0.64	0.65	0.91	0.91	0.91	0.88	0.72
S‐PLM	0.25	0.14	0.38	0.82	0.55	0.56	**0.92**	0.91	0.90	0.88	0.65
S‐PLM & ESM2‐650M	0.4	0.37	0.3	0.85	0.58	0.62	0.91	**0.92**	0.91	0.89	0.52
ESM2‐650M	0.3	0.43	0.28	0.85	0.59	0.65	0.91	0.91	**0.93**	**0.90**	0.48

^a)^ Results are directly obtained from Table 2 of the PROBE^[^ ^43^ ^]^ paper. The performance of representation methods on each task is shown with average scores. The best results for each task are shown in bold.

The benchmarking results indicate that ProtT5‐XL consistently outperformed other methods across multiple tasks, benefiting from its large encoders with 1.2B parameters. In contrast, S‐PLM & ESM2‐650M, offered balanced performance across various tasks. ProstT5, built on the ProtT5‐XL base (1.2B parameters), ranked second in the ontology‐based PFP benchmarks. PromptProtein with 650M parameters did not achieve the best performance in any of the subtasks. Our S‐PLM alone performed well, particularly in drug target PFC, matching top models like ProtT5‐XL and ProtALBERT, likely due to its structure‐aware capabilities. However, S‐PLM struggled with PPI binding affinity estimation (MSE: 0.65), which was worse than PromptProtein (MSE: 0.55) but better than ProstT5 (MSE: 0.72), possibly because its protein‐level contrastive learning is less sensitive to residue‐level mutations. When comparing S‐PLM with its base model ESM2‐650M, S‐PLM only outperformed in one SSI task and one drug target PFC task. Similarly, ProstT5 generally underperformed compared to its base model (ProtT5‐XL), except for a slight improvement in the CC category of the SSI task. This suggests that while the structure‐aware enhancements in both ProstT5 and S‐PLM are valuable, they might slightly compromise sequence representation performance. Interestingly, the combination of S‐PLM & ESM2‐650M often balances and enhances the strengths of both models. For instance, in SSI, the combined model achieves higher correlations (e.g., 0.4 in MF) than S‐PLM alone and ESM2‐650M, and in drug target PFC, it maintains a high MCC, showcasing robust performance across different datasets.

### 2.8 Ablation Study

In designing S‐PLM, we explored various structure encoders, sequence encoders, and pretraining strategies. This section summarizes key findings from these explorations.

Given that the contact map representation resembles an image, we considered several well‐established image‐processing networks as potential encoders. Specifically, we evaluated ResNet50,^[^ ^44^ ^]^ the Segment Anything Model (SAM),^[^ ^45^ ^]^ and the Swin‐Transformer^[^ ^28^ ^]^ models due to their prominence in image‐related tasks and strong performance in various competitions. Using pretrained weights, we assessed these models on CATH domains with known protein structures. As shown in Figure S2 (Supporting Information), the Swin‐Transformer (swinv2‐tiny‐patch4‐window8‐256) exhibited superior feature extraction capabilities from the contact map representation, leading us to select it as the structure encoder for S‐PLM.

For the sequence encoder, we tested various architectures and pretraining strategies by conducting preliminary training sessions, each lasting 10 000 steps. We used a consistent batch size of 20 and trained each model variation with three different random seeds to ensure robustness. The hyperparameter *K* was used to control the number of top Transformer layers of the ESM2 base model into which the adapter module is inserted, as well as the number of layers fine‐tuned or tuned using LoRA. We evaluated the performance using three protein clustering tasks: CATH clustering, deaminase clustering, and kinase group clustering. The Adjusted Rand Index (ARI) was calculated for each task, and an average ARI score was derived to provide an overall assessment. For adapter tuning, although we initially explored larger values of *K*, we ceased further attempts after observing a performance decline at higher *K* values. For the fine‐tuning strategy, the number of trainable parameters is significantly larger, which limited our ability to experiment with higher values of *K*. As a result, we only explored values up to 4. In contrast, for LoRA tuning, we could attempt much larger values of *K*, but since the base model has a maximum of 33 layers, *K* could not exceed 33.

The results are presented in Figure S3 (Supporting Information). The results show that increasing *K* for adapter tuning generally improved clustering performance in the CATH task, particularly for the CATH Class and Topology categories. However, this improvement came at the cost of reduced performance in the other two tasks. This outcome is expected, as a higher number of adapter layers exerts a greater influence on the base model, making the Structural‐Aware Module more effective in learning structural features but potentially compromising the base model's sequence representation performance. Fine‐tuning with *K*  =  4 achieved moderate improvements in all tasks, but it did not match the performance gains seen with higher *K* values in adapter tuning. LoRA tuning with larger *K* values showed competitive performance in the CATH clustering tasks. However, similar to adapter tuning, there was a trade‐off in performance for the other clustering tasks, particularly when *K* approached the upper limit. Based on these findings, we ultimately selected adapter tuning with *K*  =  16 for our pretraining, balancing the gains in structural feature learning with the preservation of sequence representation performance.

## 3 Discussion and Conclusion

In this work, we proposed S‐PLM, a structure‐aware protein language model pretrained via contrastive learning between sequence and 3D structure of proteins. Distinguishing from joint‐embedding‐based methods, S‐PLM independently encodes protein sequences and 3D structures. This unique feature allows S‐PLM to make structure‐aware predictions using protein sequences only without its 3D structure. This aspect is particularly important when obtaining reliable protein structure is difficult or time‐consuming. By employing a Struct‐Aware Module on the pretrained ESM2 model, the sequence encoder of S‐PLM generates sequence representations that seamlessly incorporate 3D structure information while maintaining the original sequence representation capabilities of ESM2. Importantly, this adapter‐based architecture is extensible; in the future, if new protein attributes need to be included, such as protein function and protein‐protein interactions, the existing ESM2 and Struct‐Aware Module can remain unchanged. New attributes can be integrated by simply adding a parallel adapter and training on updated data, as illustrated in Figure 1c.

The results of our study highlight the efficacy of S‐PLM across a range of downstream use cases. First, S‐PLM demonstrates the ability to align sequence and structure embeddings of the same protein effectively while keeping embeddings from other proteins further apart. Secondly, compared to other PLMs that only use sequences, S‐PLM shows impressive awareness of protein structures, as evidenced by its superior performance in clustering CATH domains and enzyme clustering tasks. Finally, together with S‐PLM model, we developed a library of lightweight training strategies that can be applied to train S‐PLM. Even without inputting protein structure, S‐PLM achieves competitive performance in protein‐supervised prediction tasks, comparable to the SOTA methods that require both sequence and structure inputs. These findings collectively highlight the potential of S‐PLM, together with its lightweight tuning toolbox, as an alternative PLM for protein analysis and prediction tasks.

We also observed that different tuning strategies exhibited varying performance across tasks (Table 2). This can be attributed to several factors. First, the number of trainable parameters plays a role, with fine‐tuning top layers generally providing more trainable parameters than adapter tuning, and LoRA having the fewest trainable parameters. However, the extent of adaptation to which the base model can be modified is also crucial. Despite having fewer trainable parameters, LoRA's ability to influence more layers of the base model may enable it to capture complex patterns better suited for certain tasks that diverge significantly from the pretraining objective. Additionally, the nature of the tasks themselves likely contributes to the performance differences. Some tasks may benefit more from fine‐grained adaptation of the lower layers, while others might require more substantial changes to the higher‐level representations. The diverse lightweight training strategies and related open‐source tools we provide offer flexibility in tailoring the number of trainable parameters and the extent of adaptation, potentially enabling SOTA performance on a wide range of downstream tasks.

In the benchmarking using the PROBE platform, we observed that when PLMs are used solely to generate protein representations or trained with a simple classifier, larger models often perform better across a wide range of tasks. Given that S‐PLM is not an exceptionally large model, it does not excel in all tasks. Another possible explanation for S‐PLM's underperformance in certain tasks compared to the ESM2 base model could be attributed to the fact that around 32% of the sequences in the PROBE datasets exceed the maximum length (512) used during our pretraining. This suggests that our structure‐aware approach may not be fully effective for longer sequences, indicating a need to increase the maximum sequence length during training, potentially up to 1024 amino acids, as is done with other PLMs. Additionally, enhancing S‐PLM's performance at the residue level will be an important area for future improvement. Furthermore, it appears no single PLM can outperform all others for all downstream tasks. This suggests the need to have multiple PLMs, each with multiple settings, to address diverse protein downstream tasks. Hence, the goal of S‐PLM is not to replace others, but to serve as one of valuable PLM options for users to choose and adapt for their tasks.

Interestingly, the combined S‐PLM & ESM2‐650M model, which merges the representations from both S‐PLM and ESM2‐650M, demonstrated balanced and often enhanced performance across various PROBE tasks. These results suggest that while S‐PLM provides specific advancements, particularly in integrating structural information, the base ESM2‐650M model remains highly valuable. Their combination leverages the strengths of both models, leading to more robust and versatile performance across a diverse set of tasks.

While S‐PLM has shown promising results using the AlphaFold2‐SwissProt database (0.5 M proteins), there is significant potential for further improvement by expanding our training to include more comprehensive protein structure repositories. Our current adapter‐tuning approach provides a strong foundation for continuous, lifelong learning. By continually refining and expanding the training dataset, we aim to equip S‐PLM with a broad understanding of protein structures across diverse biological contexts. This iterative process of data augmentation and model refinement holds promise for pushing the boundaries of sequence‐based protein representation learning, allowing S‐PLM to achieve even greater efficacy in a broader range of protein analysis and prediction tasks.

## 4 Experimental Section

### Sequence Encoder

Our sequence encoder was developed based on the pretrained ESM2 model.^[^ ^5^ ^]^ Given the constraints of computational resources and model capacity, ESM2‐t33_650M_UR50D as our base PLM model was selected, which had 650 million parameters. In particular, the input protein sequence was first tokenized by one‐hot encoding for each amino acid and then applied 33 layers of Transformer encoders. The embedding dimension for each position was 1280. In this procedure, a BEGIN token (“<cls>”) and END token (“<eos>”) were added to the sequence and went through the Transformer together with the amino acid tokens, and a “<pad>” token was used for padding sequences. Through the Transformer layers, the output was a tensor of 1280D vector per residue, with the embedding of the BEGIN and END tokens as well as the embeddings for padding sequences. The embeddings for each residue were used for residue‐level representations of a protein for downstream tasks. The average of per‐residue embeddings, excluding the padding tokens, was used for protein‐level embedding for the contrastive‐learning training and downstream tasks. Then, two projector layers were applied to the protein‐level embedding that transformed the dimension into the final output protein‐level embedding, which was 256D. The projectors were implemented using two dense neural network layers, with the output dimension of 256.

To optimize training efficiency with batch training while conserving GPU memory, input sequences longer than 512 residues were truncated. For protein representation generation, a parameter was provided that allowed the user to choose between using the full‐length sequence or applying truncation. For downstream tasks requiring further training, protein sequences were truncated according to task‐specific maximum lengths. The exact truncation lengths are detailed in the corresponding task section and can be configured through the task‐specific configuration files.

### Structure Encoder

The contact map was utilized to represent the 3D protein structure because it contains complete protein structure information, possesses inherent invariance capabilities, and was straightforward for implementation. Therefore, our structure encoder was specifically designed to encode the protein contact maps. Because the contact map representation resembles an image, any widely adopted networks for image‐related takes can be employed as the encoder. ResNet50,^[^ ^44^ ^]^ Segment Anything Model (SAM),^[^ ^45^ ^]^ and Swin‐Transformer^[^ ^28^ ^]^ models were considered due to their popularity as image networks and superior performance in various competitions. By evaluating them with pretrained weights on the CATH domains with known protein structures (Figure S2, Supporting Information), the Swin‐Transformer (swinv2‐tiny‐patch4‐window8‐256) was finally applied because it enables more effective feature extraction from the contact map representation. To meet the requirement of the image network, which expects three input channels, the contact map was transformed into a representation with three channels. The raw contact map was generated by calculating the coordinate distance between the C_α_ atoms for each amino acid for one sequence. In general, a contact map was a binary matrix with a value of 1 if the pairwise distance was within a chosen threshold, indicating contact between the residues; otherwise, its assigned value was 0.

In our case, a distance threshold was applied for each channel and converted the raw contact map into a continuous similarity matrix. Specifically, the distance threshold for each channel was *d*: 22Å, the same value used in AlphaFold2. The value of the similarity matrix ranges from 0 to 1, with 1 indicating the shortest pairwise distance, and 0 indicating the longest distance. The final contact map representation can be formulated as the following:

(1) $$\left(d-CLIP\left(C,d\right)\right)/d$$

where *C* represents the element of the raw contact map, and CLIP was a function that can change the *C* values higher than *d* into *d*. By averaging the embeddings of the representation layer of Swin‐Transformer and excluding the padding representation, the protein‐level embedding of structure was obtained for the contrastive‐learning training. Then, two projector layers were applied to the protein‐level embedding to transform the dimension into the final output protein‐level embedding, which was 256D, the same as the final output protein‐level embedding from sequence.

### Multi‐View Contrastive Learning

The objective of contrastive learning in this study was to bring closer the sequence embeddings and structure embeddings from the same protein and further repel all the embeddings from different proteins in the latent space. To achieve this, a multi‐view contrastive loss function was applied to the protein‐level embeddings obtained from the last projection layer of the sequence and structure encoders. The multi‐view contrastive loss function was modified based on the *NT‐Xent* (the normalized temperature‐scaled cross‐entropy loss) in SimCLR.^[^ ^29^ ^]^ In contrast to the SimCLR paper, in our approach, the positive pair was only defined for sequence embedding ($E_{seq}^{i}$) and structure embedding ($E_{str}^{i}$) from the same protein *i*. Then the multi‐view contrastive loss function for the positive pair ($E_{seq}^{i}$, $E_{str}^{i}$) is defined in the following equation:

(2) $$L_{E_{seq}^{i},\;E_{str}^{i}}=-\log\frac{\exp\left(\frac{sim\left(E_{seq}^{i},E_{str}^{i}\right)}{\tau }\right)}{\sum_{k=1}^{N}1_{k\neq i}\exp\left(\frac{sim\left(E_{seq}^{i},E_{str}^{k}\right)}{\tau }\right)+1_{k\neq i}\exp\left(\frac{sim\left(E_{seq}^{i},E_{seq}^{k}\right)}{\tau }\right)+1_{k\neq i}\exp\left(\frac{sim\left(E_{str}^{i},E_{str}^{k}\right)}{\tau }\right)}$$

where $1_{k\neq i}\in \left\{0,1\right\}$ is an indicator function equaling 1 if k≠i and *τ* denotes a temperature parameter; *N* is the batch size. The *sim*(*x*, *y*) is a function that quantifies the similarity between embeddings *x* and *y*, defined as follows:

(3) $$sim\;\left(x,y\right)=\frac{x^{T}y}{\left\|x\right\|\left\|y\right\|}$$

This loss function helps to maximize the alignment of the embeddings for protein *i* in the two views ($E_{seq}^{i},\;E_{str}^{i}$) and minimize the alignment between protein *i* and the other proteins (k≠i). The final contrastive loss was calculated for all positive pairs within a mini‐batch of samples.

### Preparation of Training Dataset for Contrastive Learning and Training Process

The training database was prepared by obtaining the protein's amino acid sequences from the Swiss‐Prot library and saving them in the FASTA format. The 3D structures of the proteins were obtained from the AlphaFold2 repository. The C_α_–C_α_ contact maps for individual proteins were determined using in‐house Python code based on the AlphaFold2 predicted 3D structures. A random selection of 500 000 proteins was made from the Swiss‐Prot library for training and 41 500 proteins selected for validation. Sequences in the validation set similar to the ones in the training set were not removed. Given the massive size of the Swiss‐Prot library (542 378 proteins in total), such similarity was negligible.

During the pretraining, input proteins larger than 512 residues were truncated, retraining only the first 512 residues for both sequence and contact map. For optimization, two separate SGD optimizers with a momentum of 0.9, were employed tailored for both the sequence and structure backbones. A cyclic learning rate schedule was applied, reducing the learning rate from 0.001 to 0 over 100 steps per cycle. The batch size was set to 20, with a weight decay of 0.0005, and no gradient accumulation was used. The temperature parameter *τ* was set to 0.05. To ensure training stability, gradient norm clipping was applied with a threshold of 1.0. Additionally, the mixed precision technique^[^ ^46^ ^]^ was utilized to accelerate training and optimize GPU memory usage in all the experiments conducted in this study. The training was performed on a single A100 GPU for more than 20 epochs using the computational facility of the University of Missouri.

### Lightweight Tuning on Sequence Encoder

The lightweight tuning strategy in this paper refers to training approaches that make specific and often parameter‐efficient modifications to a preexisting model, reducing the computational resources and memory needed compared to training a model from fully fine‐tuning. The fine‐tuning top layers, LoRA, and adapter tuning were implemented on the sequence encoder of S‐PLM, as shown in Figure 1, for downstream protein sequence prediction tasks. The details for each strategy are as follows:

### Lightweight Tuning on Sequence Encoder—Fine‐Tuning Top Layers

The ESM2 backbone model had 33 total Transformer layers. Here, “fine‐tuning top layers” refers to fine‐tuning only the top *K* ≤ 33 transformer layers and freezing the remainder. Here, *K* was a hyperparameter in our configuration.

### Lightweight Tuning on Sequence Encoder—LoRA

LoRA refers to low‐rank adaptation,^[^ ^23^ ^]^ which freezes the pretrained model weights and injects trainable rank decomposition matrices into each layer of the Transformer architecture. It can greatly reduce the number of trainable parameters for downstream tasks. For a pretrained weight matrix $W_{0}\in R^{dxk}$, its update Δ*W* can be represented as a low‐rank decomposition $\Delta W=BA,\;\mathrm{where}\;B\in R^{dxr},\;A\in R^{rxk},$ and the rank r≪min(d,k). During training, *W*_0_ is frozen, *A* and *B* contain trainable parameters. For an input *x*, the LoRA forward pass yields the following:

(4) $$h=W_{0}+\Delta Wx=W_{0}+BAx$$

In the implementation of LoRA, Δ*Wx* is scaled by $\frac{\alpha }{r}$, where α is a constant in *r*. In our implementation, the low‐rank decomposition matrices were applied to the query, key, value, and output projection matrices in the self‐attention module of the top‐*K* Transformer layers of ESM2 (Figure 1d). Here, *K*, α, and *r* were hyperparameters in our configuration.

### Lightweight Tuning on Sequence Encoder—Adapter Tuning

Adapter tuning involves integrating adapter modules into the Transformer layer of the ESM2 model. The adapter modules were implemented according to Houlsby's study.^[^ ^21^ ^]^ It was positioned twice in one Transformer layer of ESM2: after the self‐attention projection and after the two feed‐forward. Each adapter module consisted of a bottleneck structure and a skip connection. The bottleneck structure compressed the input data into a reduced‐dimensional space and then reconstructed the data to restore it to the original input dimension. The bottleneck structure enabled the adapter module to have few parameters relative to the attention and feed‐forward layers in the original Transformer. The integration of the ESM2 model with the Structure‐Aware Modules is illustrated in Figure 1b. Unlike the original adapter tuning,^[^ ^21^ ^]^ which applied the adapter modules into all Transformer layers, these were specifically inserted into the top‐*K* Transformer layers of ESM2. In our configuration, *K* is a hyperparameter.

For supervised downstream tasks, adapter tuning was implemented by integrating an additional set of parallel adapters into the Structure‐Aware Modules, with each adapter dedicated to a specific task, as depicted in Figure 1c. Each of these adapter modules shared the same architecture as those in the Structure‐Aware Module and captured different aspects of the input features tailored to various downstream tasks. These modules independently processed the same input features, extracting unique representations that were then combined. For training a new downstream task, a new trainable adapter module was added to the list of parallel adapters, while all previously added parallel adapters were frozen. The process for merging the parallel adapters is detailed in Equations (5) and (6):

(5) $$h^{\prime }=h+\sum_{i=1}^{N}a_{i}\left(h\right)$$

(6) $$a\;\left(h\right)=W_{up}\;\cdot \sigma \left(W_{down}\cdot h+b_{down}\right)+b_{up}$$

where *h* represents the input features for a Transformer layer, *N* is the number of parallel adapters and *a_i_* (·) denotes a single adapter module with *W_up_ and W_down_* as trainable projection matrices for up‐projection and down‐projection, respectively, and the ReLU activation function 𝜎, along with trainable biases *b_up_* and *b_down_*, were also part of the adapter module.

In our experiments, which involved only a single task, *N* was set to 2, representing the Struct‐Aware Module plus one parallel adapter for task‐specific tuning.

The hyperparameter used and the number of trainable parameters for each training strategy for all the downstream tasks is shown in Table 2.

### Data Preprocessing for CATH Superfamily Clustering

The sequence data was downloaded with CATH annotations from the CATH database (release v4_3_0)^[^ ^31^ ^]^ and the cath‐dataset‐nonredundant‐S40‐v4_3_0 dataset, whose proteins of maximally 40% sequence identity were used. Only sequences that had records of known protein structures were retained. One represented sequence with the longest sequence length was selected from one CATH superfamily. The three most represented categories of class (1553 sequences), the five most represented categories of architecture (1,049 sequences), and the five most represented categories of topology (306 sequences) from the CATH hierarchy were considered. The selected CATH sequences are provided in Data S1 (Supporting Information).

### Downstream Supervised Protein Prediction Tasks

The downstream supervised protein prediction tasks included four protein‐level prediction tasks—protein fold classification, enzyme reaction classification, GO term prediction, and EC number prediction—and one residue‐level prediction task, protein secondary structure prediction. For protein‐level tasks, mean pooling was applied to obtain protein‐level representations, while for the residue‐level task, the residue‐level representations were directly used as generated by the sequence encoder. A task‐specific classification layer was then applied to the corresponding representations.

### Downstream Supervised Protein Prediction Tasks—Fold Classification

Protein fold prediction was utilized to accurately determine the fold class label of a given protein. The same dataset splits from the study were used.^[^ ^47^ ^]^ The dataset comprised 16 712 proteins, classified into 1195 distinct fold classes. The dataset has three test sets: “Fold,” “Superfamily,” and “Family.” In the “Fold” set, training was conducted, excluding proteins from the same superfamily. In the “Superfamily” set, proteins from the same family were excluded from training. In the “Family” set, proteins belonging to the same family were included in the training process. Input sequences exceeding 512 residues were truncated, retaining only the first 512 residues for each sequence. It was formulated as a multi‐class classification problem. A dense neural network layer with an output dimension of 1195 was used as the classification layer, and focal loss^[^ ^48^ ^]^ (gamma = 2.0) was applied to the output.

### Downstream Supervised Protein Prediction Tasks—Enzyme Reaction Classification

The goal of enzyme reaction classification was to determine the class of enzyme‐catalyzed reactions for a protein, utilizing all four levels of EC numbers.^[^ ^49^ ^]^ Following the methodology described in the study,^[^ ^50^ ^]^ the dataset was split into training, validation, and test sets, comprising 37 428 proteins categorized across 384 four‐tiered EC numbers. All proteins had less than 50% sequence similarity across the data splits. Input sequences exceeding 512 residues were truncated, retaining only the first 512 residues for each sequence. It was formulated as a multi‐class classification problem. A dense neural network layer with an output dimension of 384 was used as the classification layer, and focal loss (gamma = 2.0) was applied to the output.

### Downstream Supervised Protein Prediction Tasks—GO Term Prediction

The objective of GO term prediction was to ascertain the association of a protein with a specific GO term, including three tasks: biological process (BP), molecular function (MF), and cellular component (CC). Each task was formulated as a multi‐label classification problem. The same dataset splits from the study were used,^[^ ^51^ ^]^ where the test set has up to 95% (30%, 40%, 50%, 70%, and 95%) sequence similarity with the training set. Input sequences exceeding 512 residues were truncated, retaining only the first 512 residues for each sequence. A dense neural network layer was used as the classification layer for each subtask, with an output dimension of 1943 for BP, 489 for MF, and 320 for CC. The binary cross‐entropy loss, implemented using ‘torch.nn.BCEWithLogitsLoss’ in Pytorch, was applied to each output.

### Downstream Supervised Protein Prediction Tasks—EC Number Prediction

The objective of this task was to identify the 538 distinct third‐ and fourth‐level EC numbers, which characterize the catalytic functions of various proteins in biochemical reactions. It was formulated as a multi‐label classification problem. The same dataset splits from the paper were used,^[^ ^51^ ^]^ where the test set has up to 95% (30%, 40%, 50%, 70%, and 95%) sequence similarity with the training set. Input sequences exceeding 512 residues were truncated, retaining only the first 512 residues for each sequence. A dense neural network layer with an output dimension of 538 was used as the classification layer. The binary cross‐entropy loss, implemented using “torch.nn.BCEWithLogitsLoss” in Pytorch, was applied to the output.

### Downstream Supervised Protein Prediction Tasks—Secondary Structure Prediction

This task aims to predict the local structures of protein residues into three secondary structure labels (i.e., coil, strand, or helix). The same dataset splits in the reference were used,^[^ ^41^ ^]^ adopting Klausen's dataset^[^ ^52^ ^]^ as the training set and the CB513 dataset^[^ ^53^ ^]^ as the test set. No two proteins have greater than 25% sequence identity, and the test set has less than 25% sequence identity against the training set. As done in the reference,^[^ ^41^ ^]^ sequences with more than 1022 residues were truncated by keeping the first 1022 residues. It was formulated as a residue‐level multi‐class classification problem. A shared dense neural network layer with an output dimension of three was used as the classification layer for each residue. The focal loss (with gamma = 2.0) was applied to the output, with padding tokens masked.

The statistics of the dataset for each task is shown in Table S2 (Supporting Information). The data can be downloaded from https://github.com/duolinwang/S-PLM/tree/main/SPLM_Data/. For all downstream training, the Adam optimizer were utilized. Detailed configuration settings can be found in the GitHub repository associated with this paper (https://github.com/duolinwang/S-PLM/tree/main/configs)

### Implementation of the Lightweight Tunning Tools

The source code of the lightweight tunning tools is available at https://github.com/duolinwang/S-PLM/. Developed primarily using PyTorch (version 1.12.1), these tools facilitate training and evaluation for the protein prediction tasks described in Methods 7. Users can reproduce the results presented in this paper by retraining with the provided configuration files in the “configs” folder. For customized training with user‐specific data, users should incorporate specific data‐processing functions into the data.py file to adapt the data format to our model. Protein‐level prediction tasks, such as fold and GO tasks, can be referenced for protein‐level prediction, while the secondary structure task serves as a reference for residue‐level prediction. Additionally, users have the flexibility to select the desired lightweight tuning strategy and modify specific parameters by adjusting the parameters in the configuration files.

## Conflict of Interest

The authors declare no conflict of interest.

## Supporting information



Supporting Information

## Data Availability

The data that support the findings of this study are available from the corresponding author upon reasonable request.
